# Supplementary material for: Machine Learning Accelerates Crystallization for Structure Determination
Source: Angew Chem Int Ed Engl. 2026 May 4;65(25):e1218503. doi: 10.1002/anie.1218503 (PMC13266958; doi:10.1002/anie.1218503)

checkCIF/PLATON report

Structure factors have been supplied for datablock(s) lcz020

THIS REPORT IS FOR GUIDANCE ONLY. IF USED AS PART OF A REVIEW PROCEDURE FOR PUBLICATION, IT SHOULD NOT REPLACE THE EXPERTISE OF AN EXPERIENCED CRYSTALLOGRAPHIC REFEREE.

No syntax errors found.   CIF dictionary   Interpreting this report

Datablock: lcz020

Bond precision:	C-C = 0.0140 A		Wavelength=1.54184
Cell:	a=8.0878(1)	b=16.6965(2)	c=24.5862(3)
	alpha=90	beta=97.799(1)	gamma=90
Temperature:	100 K		

	Calculated	Reported
Volume	3289.36(7)	3289.36(7)
Space group	P 21/n	P 1 21/n 1
Hall group	-P 2yn	-P 2yn
Moiety formula	C25 H15 Ag3 F18 N6 O3	C15 H3 Ag3 F18 N6, C10 H12 O3
Sum formula	C25 H15 Ag3 F18 N6 O3	C25 H15 Ag3 F18 N6 O3
Mr	1113.04	1113.04
Dx,g cm-3	2.248	2.248
Z	4	4
Mu (mm-1)	15.538	15.538
F000	2136.0	2136.0
F000'	2146.42	
h,k,lmax	10,21,31	9,20,31
Nref	7036	6724
Tmin,Tmax	0.112,0.045	0.537,1.000
Tmin'	0.018	

Correction method= # Reported T Limits: Tmin=0.537 Tmax=1.000
AbsCorr = MULTI-SCAN

Data completeness= 0.956 Theta(max)= 77.890

R(reflections)= 0.0684(5861) wR2(reflections)= 0.1853(6724)

S = 1.055 Npar= 499

The following ALERTS were generated. Each ALERT has the format
test-name_ALERT_alert-type_alert-level.
Click on the hyperlinks for more details of the test.

Alert level C

```
PLAT042_ALERT_1_C Calc. and Reported MoietyFormula Strings  Differ     Please Check
              Calc: C25 H15 Ag3 F18 N6 O3
              Rep.: C15 H3 Ag3 F18 N6, C10 H12 O3
PLAT213_ALERT_2_C Atom F00I            has ADP max/min Ratio .....        3.1 prolat
PLAT213_ALERT_2_C Atom F01D            has ADP max/min Ratio .....        3.3 prolat
PLAT213_ALERT_2_C Atom F01E            has ADP max/min Ratio .....        3.3 prolat
PLAT213_ALERT_2_C Atom F01I            has ADP max/min Ratio .....        3.2 prolat
PLAT213_ALERT_2_C Atom C01G            has ADP max/min Ratio .....        3.1 prolat
PLAT220_ALERT_2_C NonSolvent   Resd 1  F   Ueq(max)/Ueq(min) Range        4.0 Ratio
PLAT241_ALERT_2_C High   'MainMol' Ueq as Compared to Neighbors of       O00Q Check
PLAT342_ALERT_3_C Low Bond Precision on  C-C Bonds ..............      0.014 Ang.
PLAT906_ALERT_3_C Large K Value in the Analysis of Variance ......      7.174 Check
PLAT906_ALERT_3_C Large K Value in the Analysis of Variance ......      2.228 Check
PLAT911_ALERT_3_C Missing FCF Refl Between Thmin & STh/L=    0.600         25 Report
               -1  0  1,  -3 19  1,   0  1  2,   2 19  2,   6  9  3,   4 10  4,
               -9  0  5,  -9  1  6,  -9  0  7,  -9  1  7,  -9  2  7,  -1 13  7,
               -9  1  8,  -9  3  8,  -9  0  9,  -2 18 11,  -8  1 13,  -8  0 14,
               -8  1 15,  -1  6 15,   0  8 15,  -8  0 16,  -8  1 17,   0  1 21,
               -1  1 25,
PLAT971_ALERT_2_C Check Calcd Resid. Dens.  0.91Ang From F01E           2.19 eA-3
PLAT971_ALERT_2_C Check Calcd Resid. Dens.  0.98Ang From F01E           1.89 eA-3
PLAT971_ALERT_2_C Check Calcd Resid. Dens.  1.09Ang From F01D           1.67 eA-3
PLAT977_ALERT_2_C Check Negative Difference Density on H00T      .      -0.33 eA-3
PLAT977_ALERT_2_C Check Negative Difference Density on H01I      .      -0.34 eA-3
```

Alert level G

```
PLAT003_ALERT_2_G Number of Uiso or U(i,j)  Restrained non-H-Atoms          4 Report
PLAT083_ALERT_2_G SHELXL Second Parameter in WGHT  Unusually Large      48.48 Why ?
PLAT186_ALERT_4_G The CIF-Embedded .res File Contains ISOR Records          1 Report
PLAT242_ALERT_2_G Low    'MainMol' Ueq as Compared to Neighbors of       C00Y Check
PLAT242_ALERT_2_G Low    'MainMol' Ueq as Compared to Neighbors of       C016 Check
PLAT242_ALERT_2_G Low    'MainMol' Ueq as Compared to Neighbors of       C01A Check
PLAT242_ALERT_2_G Low    'MainMol' Ueq as Compared to Neighbors of       C01B Check
PLAT242_ALERT_2_G Low    'MainMol' Ueq as Compared to Neighbors of       C01F Check
PLAT242_ALERT_2_G Low    'MainMol' Ueq as Compared to Neighbors of       C01G Check
PLAT720_ALERT_4_G Number of Unusual/Non-Standard Labels ..........         70 Note
              Ag01    Ag02    Ag03    F004    F005    F006    F007    F008
              F009    F00A    F00B    F00C    F00D    F00E    F00F    N00G
              N00H    F00I    N00J    N00K    N00L    F00M    O00N    N00O
              O00P    O00Q    C00R    H00R    C00S    H00S    C00T    H00T
              C00U    C00V    C00W    C00X    C00Y    C00Z    H00Z    C010
```

```
             C011    C012    H012    C013    C014    C015    H01A    H01B
             H01C    C016    C017    C018    H018    C019    C01A    C01B
             C01C    H01D    H01E    H01F    F01D    F01E    C01F    C01G
             C01H    H01G    H01H    H01I    F01I    F01J
PLAT860_ALERT_3_G Number of Least-Squares Restraints ............         24 Note
PLAT883_ALERT_1_G Absent Datum for _atom_sites_solution_primary ..     Please Do !
PLAT912_ALERT_4_G Missing # of FCF Reflections Above STh/L=  0.600        227 Note
PLAT933_ALERT_2_G Number of HKL-OMIT Records in Embedded .res File          2 Note
               0  1 21,  -1  1 25,
PLAT941_ALERT_3_G Average HKL Measurement Multiplicity ..........         2.5 Low
PLAT969_ALERT_5_G The 'Henn et al.' R-Factor-gap value ..........       3.259 Note
             Predicted wR2: Based on SigI**2  5.69 or SHELX Weight 17.57
PLAT978_ALERT_2_G Number C-C Bonds with Positive Residual Density.          0 Info
```

```
   0 ALERT level A = Most likely a serious problem - resolve or explain
   0 ALERT level B = A potentially serious problem, consider carefully
  17 ALERT level C = Check. Ensure it is not caused by an omission or oversight
  17 ALERT level G = General information/check it is not something unexpected

   2 ALERT type 1 CIF construction/syntax error, inconsistent or missing data
  22 ALERT type 2 Indicator that the structure model may be wrong or deficient
   6 ALERT type 3 Indicator that the structure quality may be low
   3 ALERT type 4 Improvement, methodology, query or suggestion
   1 ALERT type 5 Informative message, check
```

Validation response form

Please find below a validation response form (VRF) that can be filled in and pasted into your CIF.

```
# start Validation Reply Form
_vrf_PLAT042_lcz020
;
PROBLEM: Calc. and Reported MoietyFormula Strings  Differ     Please Check
RESPONSE: ...
;
_vrf_PLAT213_lcz020
;
PROBLEM: Atom F00I            has ADP max/min Ratio .....        3.1 prolat
RESPONSE: ...
;
_vrf_PLAT220_lcz020
;
PROBLEM: NonSolvent   Resd 1  F   Ueq(max)/Ueq(min) Range        4.0 Ratio
RESPONSE: ...
;
_vrf_PLAT241_lcz020
;
PROBLEM: High   'MainMol' Ueq as Compared to Neighbors of       O00Q Check
RESPONSE: ...
;
_vrf_PLAT342_lcz020
;
PROBLEM: Low Bond Precision on  C-C Bonds ..............      0.014 Ang.
RESPONSE: ...
;
```

```
_vrf_PLAT906_lcz020
;
PROBLEM: Large K Value in the Analysis of Variance ......      7.174 Check
RESPONSE: ...
;
_vrf_PLAT911_lcz020
;
PROBLEM: Missing FCF Refl Between Thmin & STh/L=    0.600         25 Report
RESPONSE: ...
;
_vrf_PLAT971_lcz020
;
PROBLEM: Check Calcd Resid. Dens.  0.91Ang From F01E            2.19 eA-3
RESPONSE: ...
;
_vrf_PLAT977_lcz020
;
PROBLEM: Check Negative Difference Density on H00T      .      -0.33 eA-3
RESPONSE: ...
;
# end Validation Reply Form
```

It is advisable to attempt to resolve as many as possible of the alerts in all categories. Often the minor alerts point to easily fixed oversights, errors and omissions in your CIF or refinement strategy, so attention to these fine details can be worthwhile. It is up to the individual to critically assess their own results and, if necessary, seek expert advice.

PLATON version of 17/09/2025; check.def file version of 17/09/2025

---

duplicate check

No duplication found

Datablock lcz020 - ellipsoid plot

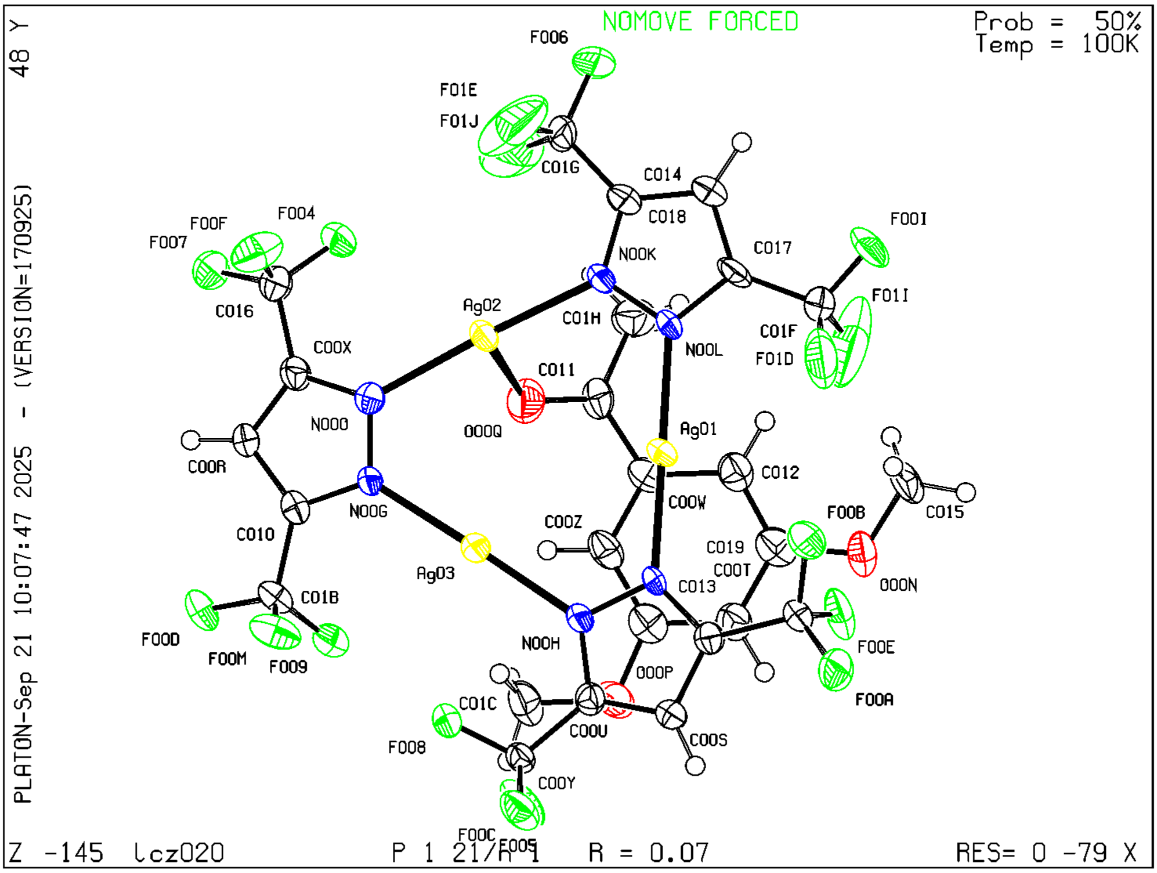

Supplement: Supplementary file 2 — Supporting File 2: anie72462‐sup‐0002‐DataFile.zip. [file ANIE-65-e1218503-s002.zip › supplementary_materials_crystallographic_data submit.zip!/cif_and_checkcif(1-114)/Ag3Pz3_20/Ag3Pz3_20_checkcif.pdf]
